# Key Components in eHealth Interventions Combining Self-Tracking and Persuasive eCoaching to Promote a Healthier Lifestyle: A Scoping Review

**DOI:** 10.2196/jmir.7288

**Published:** 2017-08-01

**Authors:** Aniek J Lentferink, Hilbrand KE Oldenhuis, Martijn de Groot, Louis Polstra, Hugo Velthuijsen, Julia EWC van Gemert-Pijnen

**Affiliations:** ^1^ Centre for eHealth & Wellbeing Research Departement of Psychology, Health, and Technology University of Twente Enschede Netherlands; ^2^ Marian van Os Centre for Entrepreneurship Hanze University of Applied Sciences Groningen Netherlands; ^3^ Quantified Self Institute Hanze University of Applied Sciences Groningen Netherlands

**Keywords:** telemedicine, review, health promotion, remote sensing technology

## Abstract

**Background:**

The combination of self-tracking and persuasive eCoaching in automated interventions is a new and promising approach for healthy lifestyle management.

**Objective:**

The aim of this study was to identify key components of self-tracking and persuasive eCoaching in automated healthy lifestyle interventions that contribute to their effectiveness on health outcomes, usability, and adherence. A secondary aim was to identify the way in which these key components should be designed to contribute to improved health outcomes, usability, and adherence.

**Methods:**

The scoping review methodology proposed by Arskey and O’Malley was applied. Scopus, EMBASE, PsycINFO, and PubMed were searched for publications dated from January 1, 2013 to January 31, 2016 that included (1) self-tracking, (2) persuasive eCoaching, and (3) healthy lifestyle intervention.

**Results:**

The search resulted in 32 publications, 17 of which provided results regarding the effect on health outcomes, 27 of which provided results regarding usability, and 13 of which provided results regarding adherence. Among the 32 publications, 27 described an intervention. The most commonly applied persuasive eCoaching components in the described interventions were *personalization* (n=24), *suggestion* (n=19), *goal-setting* (n=17), *simulation* (n=17), and *reminders* (n=15). As for self-tracking components, most interventions utilized an accelerometer to measure steps (n=11). Furthermore, the medium through which the user could access the intervention was usually a mobile phone (n=10). The following key components and their specific design seem to influence both health outcomes and usability in a positive way: *reduction* by setting short-term goals to eventually reach long-term goals, *personalization* of goals, *praise* messages, *reminders* to input self-tracking data into the technology, use of *validity-tested* devices, *integration of self-tracking and persuasive eCoaching*, and provision of face-to-face instructions during *implementation*. In addition, health outcomes or usability were not negatively affected when more *effort* was requested from participants to input data into the technology. The data extracted from the included publications provided limited ability to identify key components for adherence. However, one key component was identified for both usability and adherence, namely the provision of *personalized* content.

**Conclusions:**

This scoping review provides a first overview of the key components in automated healthy lifestyle interventions combining self-tracking and persuasive eCoaching that can be utilized during the development of such interventions. Future studies should focus on the identification of key components for effects on adherence, as adherence is a prerequisite for an intervention to be effective.

## Introduction

### Health Promotion and Technology

Improving healthy lifestyle behavior is an effective strategy to decrease mortality and increase health-related quality of life [1,2]. Current digital health technologies provide meaningful contributions to the design of healthy lifestyle interventions and the dissemination of such interventions [3]. A combination of self-tracking, goal-setting, and feedback in automated interventions has been indicated by many to be an effective approach for increasing healthy lifestyle behavior [3-5]. Self-tracking is “the practice of systematically recording information about one’s diet, health, or activities, typically by means of a mobile phone, so as to discover behavioral patterns that may then be adjusted to help improve one’s physical or mental well-being” [6]. Components that might be important for self-tracking are the self-tracking device, validity, the effort required of the participant to perform self-tracking, and the presentation of summary data to the user [7].

### Persuasive eCoaching

Goal-setting and feedback are components that can be provided via so-called persuasive eCoaching. This new term is a contraction of the terms “persuasive technology” and “eCoaching.” We refer to persuasive eCoaching as the use of technology during coaching to motivate and stimulate (groups of) people to change attitudes, behaviors, and rituals [8]. Oinas-Kukkonen and Harjumaa’s persuasive system design (PSD) model [9] describes such persuasive technologies that are expected to positively influence health behavior change. This PSD model builds upon earlier research by Fogg [10] and divides the persuasive components into 4 main categories: primary task support, dialogue support, system credibility support, and social support. These categories contain additional components such as personalization and reminders. To make the PSD model more complete for persuasive eCoaching, some coaching components that can be provided via technology can be added, namely educational coaching, goal-setting, and feedback.

### New Opportunities and Challenges

The integration of self-tracking and persuasive eCoaching in fully automated healthy lifestyle interventions creates new opportunities for healthy lifestyle management. First, self-tracking devices enable the objective tracking of lifestyle behavior such as physical activity, heart rate, or sleep. This objective measurement of one’s lifestyle pattern can be more reliable than people’s own estimations based on their memory and biological sensing of their lifestyle patterns [11-13]. More reliable measurements could become an essential component in lifestyle behavior change, enabling a greater awareness of people’s current lifestyles [14]. Second, data from wearable devices can generate automated, personally relevant feedback 24/7. Previous research suggests that this just-in-time tailored feedback contributes to the sustainable use of the intervention [8,15,16]. Third, more and more people own devices that are suitable for eHealth interventions [3]. Even among ethnic minorities and the elderly, the use of mobile phones and computers is rising [17-19]. This suggests a certain scalability for such interventions and maybe even cost-effectiveness due to the fact that no human effort is required to carry them out.

Besides these opportunities, applying the combination of self-tracking and persuasive eCoaching in automatic eHealth interventions also gives rise to a few challenges. These challenges concern privacy issues, trust, and ethics due to personally sensitive data being obtained and stored [20-22]. Concerning ethics, suggestions based on self-tracking data that are invalidated or unsupervised might end up being incorrect or even harmful [21]. In addition, individuals need to be able to understand and interpret the self-tracking data [23].

### Identifying Key Components

Despite the challenges, the combination of self-tracking and persuasive eCoaching to promote a healthier lifestyle is promising [3-5], and consequently, interventions employing this combination are becoming more common [5]. To our knowledge, no literature review has been conducted to identify the key components of such interventions. Knowledge about these key components can serve as input for future development of healthy lifestyle interventions that combine self-tracking and persuasive eCoaching, which in turn might increase the effect on health outcomes, usability, and adherence. Usability and adherence are important effect measures of eHealth interventions as they are prerequisites for the intervention to positively influence health or health behavior. In addition, it is worthwhile to identify the specific way a key component should be designed to create positive effects on health outcomes, usability, and adherence. “Effect on health outcomes” here means the effects of the lifestyle intervention on both changes in healthy lifestyle behavior (eg, an increase in physical activity) as well as changes in health status (eg, improved blood levels or weight loss). “Usability” here means the user’s satisfaction with the technology and its ease of use [24]. “Adherence” here means the extent to which the technology is used as intended [15].

Key components of interest are self-tracking components (eg, type of device and presentation of summary data to the user), persuasive eCoaching components (eg, elements of the PSD model such as personalization and suggestion), and other intervention components (eg, the underlying behavior change theory and cocreation with end users). This review addresses the following research questions: (1) What are key components for the effectiveness on health outcomes, usability, and/or adherence of automated healthy lifestyle interventions combining self-tracking and persuasive eCoaching? and (2) In which way should key components be designed to contribute to effectiveness on health outcomes, usability, and/or adherence?

### Overarching Project

This review is part of an overarching project for the development of a workplace stress management intervention that combines self-tracking and persuasive eCoaching. To ensure systematic and holistic development and implementation of the eHealth intervention, the Center for eHealth Research (CeHRes) roadmap is adhered to throughout this project [8]. This evidence-based roadmap aims to improve the uptake and impact of eHealth technologies and is based on a participatory development approach, persuasive design techniques, and business modeling. The first step consists of contextual inquiry. This step aims to identify key components from the literature and from users and other stakeholders who will affect—or will be affected by—the intervention.

## Methods

### Scoping Review Methodology

As technology continues to evolve rapidly, this particular scoping review methodology was chosen for this review study because it allowed us to obtain a quick overview of the current literature on the topic. The fact that this field is rapidly evolving is illustrated by the development of Fitbit self-tracking devices. Ever since the first Fitbit tracker was released at the end of 2009, 13 more Fitbit trackers have been released [25].

Another reason to conduct a scoping review on this topic is that a scoping review is not limited to randomized controlled trials (RCTs) [26,27]. To properly identify the scope of this topic, studies evaluating the effect on health outcomes, usability, and adherence are required. Studies regarding the latter two will primarily be qualitative studies [24].

Arksey and O’Malley’s scoping review methodology [26] was applied. This methodology comprises the following steps: (1) identifying the research question; (2) identifying relevant studies; (3) study selection; (4) charting the data; (5) collating, summarizing, and reporting the results; and (6) consultation. A number of additional recommendations by Levac et al [27] were followed, namely: providing a clear purpose for the scoping review, review of full-text articles by 2 independent reviewers to decide on their inclusion, collectively developing the data-charting form with the research team, continually extracting data and updating the data-charting form, inclusion of the consultation step (an optional step according to Arksey and O’Malley [26]), and providing a clear consultation purpose.

### Identifying Relevant Studies and Study Selection

A systematic literature search was performed in PubMed, EMBASE, PsycINFO, and Scopus covering the period from January 1, 2013 to January 31, 2016. PubMed and EMBASE were chosen for their wide coverage of scientific journals, whereas PsycINFO was chosen for its specific relevance to this review’s subject. Scopus was searched because of its multidisciplinary scope, which allows for identification of articles outside the medical field, such as in engineering. We decided to include no publication from before 2013 as technologies described in publications before 2013 seem less comparable with technologies described in newer publications. To illustrate, publications containing the search terms “Fitbit” and “smartwatch” increased from a negligible number before 2013 to hundreds from 2013 onward, reflecting the rise of personal monitoring devices [28]. These personal monitoring devices represent newer self-tracking technologies that simplify the collection and combining of personal data and enable more personalized healthy lifestyle interventions [29]. Including older publications, in which technological advances are not displayed, might lead to less relevant findings [30].

This study’s search strategy was created in collaboration with a University of Twente librarian, based on 3 main components: (1) self-tracking, (2) persuasive eCoaching, and (3) healthy lifestyle interventions. Related search keywords were identified using MeSH and EMTREE terms, PubReMiner, synonyms, keywords from relevant articles, and self-determined search terms (see Multimedia Appendix 1).

Our aim was to include articles that described fully automated interventions. However, we found that many articles involved a fully automated intervention in addition to human coaching, which we call blended coaching. As the scoping review methodology allows for post hoc decisions [27], we then decided to also include blended coaching interventions because we expected to find relevant results in these studies. Other inclusion criteria were that the articles had to be written in English or Dutch and had to be journal articles. Excluded publications included reviews, study protocols, study populations outside the age range of 18-66 years, publications lacking empirical data, and paper-based or personally reported tracking. This age range is in line with the target group of our overarching research project that focuses on the working population. In a lot of European countries, the retirement age is gradually increasing toward 67 years [31].

The results of the search query were uploaded into the EndNote X7 reference manager (Thomson Reuters, Philadelphia, PA, USA) and independently assessed by two reviewers to decide on their inclusion based on title, abstract, and full-text (the review team was comprised of AL and HO for selection based on title and abstract, and AL, HO, LP, and MG for selection based on full-text, with AL reviewing all full-text articles). Differences were fully discussed until consensus was reached.

In addition to the electronic database search, manual searching was performed in *JMIR mHealth and uHealth* for issues dated from January 1, 2013 to January 31, 2016. In addition, a check was performed on the bibliographic reference lists of publications that remained after full-text selection of the search query or manual searching and did not describe interventions involving blended coaching, to identify any additional eligible publications.

**Figure 1:**
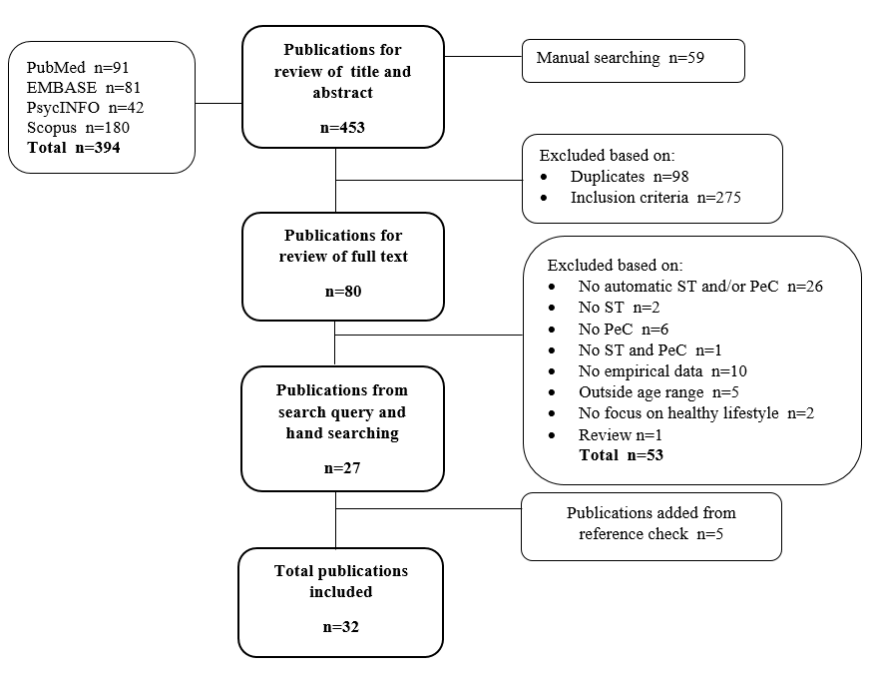
Flowchart of the selection process. Note: ST=self-tracking, PeC=Persuasive eCoaching.

The electronic database search and manual searching resulted in 394 publications and 59 publications, respectively, 98 of which were duplicates. After the final full-text selection, 27 publications remained [32-58]. The check of the reference lists resulted in 5 additional publications [59-63] (see the flowchart in Figure 1).

### Charting the Data

A data-charting form was created by the research team that included the following: study characteristics (eg, title, participants, outcomes of interest, and effectiveness), intervention characteristics (eg, short description of the intervention, self-tracking components, and persuasive eCoaching components), and advantages and limitations of the intervention and research according to the authors or reviewers (see Table 1). Next, the data-charting form was improved by several iterations between researchers and 2 consensus meetings of the whole research team.

**Table 1:** Components of the data-charting form.

Category	Component	
Study characteristics	Title	
	Author (Year)	
	**Study set-up**	
		comparison intervention
		study quality
	Objective study	
	Participants	
	Country of the study	
	Duration	
	Outcomes of interest	
	Secondary outcomes	
	Measuring instruments	
	Validity of measuring instruments	
	Effect on health outcomes (high effective, low effective, and ineffective)	
	Usability	
	Adherence	
Intervention characteristics	Intervention setting (lifestyle, chronic disease, or mental health)	
	Country of the intervention	
	**Persuasive eCoaching components**	
		components from the PSD model
		social support in general
		educational coaching
		goal-setting
		feedback
	**Self-tracking components**	
		in general
		device
		measurement variable
		the participant’s effort
		presentation of summary data
		duration of device usage
		device placement
		validity
	**Other intervention components**	
		short description of the technology
		the intervention’s aim
		theory applied
		results from other research applied
		cocreation
Advantages and limitations according to author or reviewer	Advantages and limitations of intervention	
	Advantages and limitations of research	

**Table 2:** Categorization of interventions by effect on health outcomes by Morrison et al [64].

Effectiveness	Criteria
High effective	The intervention led to statistically significant improvement on the majority of outcome measures.
	The intervention was more effective or as effective as comparison groups.
	The intervention was more effective than control groups without an intervention or waiting lists.
Low effective	The intervention led to statistically significant improvement on the minority of outcome measures.
	The intervention was as effective or less effective than comparison groups.
	The intervention was more effective than control groups without an intervention or waiting lists.
Ineffective	The intervention led to no statistically significant improvements on any of the outcome measures.
	The intervention was no more effective than control groups without an intervention or waiting lists.

Using the framework by Morrison et al [64], the interventions were divided into 3 categories in terms of their effect on health outcomes: high effective, low effective, and ineffective (see Table 2). For example, if the intervention group showed statistically significant improvements for steps per day and body mass index but not for blood pressure as a result of within-group analyses and was more effective than the comparison intervention group as a result of between-group analysis, the intervention was categorized as “high effective.” No distinction has been made between the different outcomes, as long as they were related to health (eg, healthy lifestyle behavior or health status). Study quality was assessed by evaluating the rigor of the study designs based on the established hierarchy of study designs [65].

Data on usability was extracted from included publications if participants in the study expressed a preference for a component or a specific component’s design that increased their level of satisfaction regarding the technology or its ease of use.

Data on adherence was extracted if the studies described the way in which participants should use the intervention and presented results on the participants’ adherence to that intended use. Originally, we intended to divide the studies into incremental categories of adherence. Unfortunately, the data extracted from the studies did not allow us to do so. Additionally, data on adherence was extracted if participants expressed an expectation that a specific component could increase their adherence in using the technology.

Persuasive eCoaching components were extracted from included publications using the PSD model [9]. Solely, persuasive components were coded when they were executed by the technology and not by human effort, which is in line with the use of the PSD model as described in the review by Kelders et al [15]. As data extraction progressed, we decided to include 3 persuasive eCoaching components in the data-charting form: *educational coaching*, *goal-setting*, and *feedback*. The reason for this decision was that these components were often described in the intervention’s design and comprised coaching strategies that could be delivered via technology. In addition, the specific reasons for allowing social support in the design were often omitted. Consequently, we could not link design elements to specific social support components in the PSD model and therefore created the component *social support in general* (see Multimedia Appendix 2 for an overview).

The self-tracking components and other intervention components were identified using qualitative analysis of the data from publications. Components were added or changed continuously as the qualitative data analysis progressed.

The consistency of the data-charting form was discussed by two reviewers (AL and HO), who focused on data extraction performed by one reviewer (AL) on 4 articles with various study designs (3 studies evaluating the effect on health outcomes and 1 study on usability) [32,35,50,53]. After their discussion, it appeared that persuasive eCoaching components and the advantages and limitations of the research and interventions were more prone to reviewer subjectivism than other components. Therefore, we decided to extract the data from 3 articles [42,46,49] gathered by two reviewers (AL and HO) independently of these components to increase consensus with regards to the data interpretation.

### Collating, Summarizing, and Reporting the Results

All relevant data was coded using the data-charting form in ATLAS.ti version 7.5 (Scientific Software Development GmbH, Berlin), a qualitative software package. In addition, short summaries were obtained from the data-charting forms to provide quick overviews. Qualitative analysis was used due to our interest in how and why components were applied and to observe patterns in the application of the components and their contribution to the effect on health outcomes and usability [66]. Following Arksey and O’Malley [26] and Levac et al [27], descriptive numerical summaries and thematic analyses were used for data analysis, resulting in an approach that is akin to a “narrative review” [26]. First, a descriptive numerical summary was used to create a numerical overview of specific self-tracking components, persuasive eCoaching components, and other components in the interventions categorized by their effect on health outcomes. Components were identified as key components if at least 50% of the interventions that showed effectiveness on health outcomes (high and low effective interventions) included the component. This 50% rule was applied to all persuasive eCoaching components, with exception of feedback, the self-tracking component *validity*, and the other intervention components, *theory applied*, *results from other research applied*, *cocreation*, *design testing*, *integration of self-tracking and persuasive eCoaching*, and *blended coaching*. Other components included in the data extraction were of a descriptive nature and could therefore not be treated as dichotomous components for which percentages could be calculated.

Second, thematic analysis was applied to obtain more insight into the various components’ specific designs and if these specific designs relate to the effectiveness of the interventions on health outcomes, usability, and/or adherence. When patterns were observed linking components and effectiveness, these components were then identified as key components. Additionally, thematic analysis directed the process of creating the data-charting form.

### Consultation

The aim of this consultation was to give meaning to and assess the applicability of the results by obtaining insight from other perspectives, beyond the research team’s own perspectives [26,27]. The consultation was carried out during the 11th International Conference on Persuasive Technology. The preliminary results of this scoping review [67] were presented and input was requested from experts in several fields during the workshop on Behavior Change Support Systems (BCSS 2016): Epic for Change, the Pillars for Persuasive Technology for Smart Societies. This consultation adjusted the scope to the way in which components are designed, to get a clear idea of how and why specific components do or do not contribute to effects on health outcomes, usability, and/or adherence.

## Results

### Characteristics of Included Studies

#### General Characteristics

Most studies were carried out in the United States [32,33,35,37,40,46,49-52,57,60,63], followed by the Netherlands [34,45,53,55,56,62].

Of the 32 included publications, 27 in total described an intervention [32,33,35,37-45,47-49,51-58,60-63], 17 of which [32,35,37,39,41,43-45,48,49,51-53,57,60,61,63] evaluated the effects of that intervention on health outcomes. Of these 17 studies, 16 were RCTs (highest level in the hierarchy of study designs [65]) and 1 was a quasi-RCT study (second highest level) [43]. In addition, 10 were categorized as high effective [32,39,41,45,48,49,51,60,61,63], 4 as low effective [32,37,44,57], and 3 as ineffective [35,43,52]. Additionally, 25 publications [32-36,38-43,46-51,54-59,62,63] included results on usability, 18 of which [32,33,35,36,38-43,48,49,51,54, 56,57,62,63] were based on people’s experiences after having used the technology and 6 of which were based on expectations [34,46,50,55,58,59]. Only 1 study addressed usability results based on experiences as well as those based on expectations [47]. As for adherence, 8 publications included information about the intended use of the intervention [40,41,45,47, 51,53,60,63], and 5 publications included information about expectations regarding components that could increase adherence [36,46,47,59,62] (see Multimedia Appendix 3 for a summary of the included publications).

### Intervention Characteristics

#### General Characteristics

Out of those publications that described the design of an intervention, 17 were developed in a healthy lifestyle setting [32,33,35,37,39,42-45,49,51,52,57,58,61-63] and 10 in a chronic disease setting [38,40,41,47,48,53-56,60]. Furthermore, about half of the interventions described included the application of a certain theory in the design. The most frequently applied theories were social cognitive theory [33,35,38,49,52], transtheoretical theory [32,35,39,52,55], and self-regulation theory [38,47,63]. Additionally, 6 studies included descriptions of cocreation with end users [42,47,52,55,56,58]. The medium most often used to execute the intervention was a mobile phone app [37,38,40,48,49,52,55,60-62]. Other mediums were a computer [35,39,43,45,54,63], a combination of computer and mobile phone for text messages (short message service, SMS) [32,41,42,44,51,57,58], a combination of computer and a mobile phone app [33,53,56], or just a mobile phone for text messages [56]. Finally, 10 interventions involved blended coaching [38,40,44,45,47,48,54-56,60].

#### Persuasive eCoaching Characteristics

The persuasive technology category from the PSD model applied most often was primary task support, followed by dialogue support. System credibility support was applied sparingly. For the most part, the identified persuasive eCoaching components were *personalization* (85%, or 23/27), *goal-setting* (74%, or 20/27), *suggestion* (70%, or 19/27), *simulation* (56%, or 15/27), and *reminders* (52%, or 14/27).

#### Self-Tracking Characteristics

Most interventions used an accelerometer for self-tracking [33,37,38,40,41,45,49,53,55-57]. Other devices used in multiple interventions were pedometers [32,35,39,44,47,48,51,54,58,60] and smart scales [48,52,63]. Five of these self-tracking devices [32,39,42,45,56] were described as tested for validity. In addition, the effort required of the participant to input data into the technology was either none, that is, automatic transfer of data [33,38,43,48,52,55,56,62,63], manually entering data [32,37,39,47-49,51,54,58,60], or uploading data [35,41,42, 45,57]. Four studies made no mention of the transmission of data to the technology [40,44,53,61]. The type of electronic data collected was usually the number of steps taken [32,33,35, 38-41,44,47,48,51,53,54,56-58,60,61]. Furthermore, data regarding weight [48,52,63], heart rate [42], and other types of physical activity outcomes was collected, such as distance [33,38,43], intensity [38,41,45,55-57], time [38,42,43,45,55, 57,60], and/or energy expenditure [43,45,49,61]. The electronic data was either presented to the participant as summary data via visual presentation in a graph, chart, or bar [33,35,37,38, 40,42,48,49,51,53,55,56,58,61,63], as summary data via a message [47], or in a life log with a list of activities [49]. Eleven interventions drew a comparison between the current behavior and the goal [33,37,38,48,51,53,55,56,58,61].

### Key Components

An overview of the key components, categorized by effect measures (health outcomes, usability, and adherence), can be found in Multimedia Appendix 4. This table also provides an overview of which studies the key components are based on. Results regarding components have not been presented if too little data was present (results regarding that component from only one study) or no clear pattern could be observed between the component and the effectiveness (on health outcomes, usability, and/or adherence). Moreover, key components were not separately described for interventions utilizing blended coaching or automatic coaching, as too little data was present or no differences were observed for the key components between the 2 types of coaching in terms of their effects. The same holds for key components from studies describing results regarding usability based on expectations, and studies based on experiences. The key components presented below are divided according to the 3 effect measures: health outcomes, usability, and adherence.

#### Key Components for a Positive Effect on Health Outcomes

##### Persuasive eCoaching Key Components

In the category of primary task support, *reduction* [32,39,41,49,51,60,63], *personalization* [32,37,39,41,45, 48,49,51,53,57,60,61], and *simulation* [37,39,48,51,53,57,61,63] were identified as key components, as they were included in at least 50% of the interventions that were effective in terms of health outcomes. As for dialogue support, *reminders* [32,37,44,51,53,57,60] and *suggestion* [32,37,39,45,48,49, 51,53,57,60,63] were identified as key components. No key components were identified in the categories of system credibility support and social support. *Goal-setting* was determined to be another key component for persuasive eCoaching [32,37,39,41,49,51,53,60,61,63] (see Table 3 for an overview).

##### Design of the Persuasive eCoaching Key Components

In studies evaluating the effect on health outcomes, the *reduction* component was designed in 1 of 3 ways: (1) setting short term goals to eventually reach long term goals [32,39,41,49,51,60,63], (2) providing low effort behavior suggestions [35,49,63], or (3) helping the user solve a problem [35]. *Personalization* was most often implemented to adjust goals or feedback [32,35,37,39,41,43,45,48,51-53,60,61,63] but not so much for the user’s ability to set technical features, such as their ability to control prompts and layout [32,57]. The personalization of feedback was mostly based on self-tracking data or reaching goals [32,39,41,43,45,48,52,53,60,61]. The *simulation* component consisted of an overview of the collected data over time in a graph [35,37,48,51,53,57,61,63] or in a message [39]. *Reminders* were usually sent daily [32,35,37,51,60] and were either task reminders regarding self-tracking [32,51,52,60] or reminders to perform health behavior [35,37,44,53,57]. As for *suggestion*, these messages were often personalized [35,37,45,49,51,63] and contained suggestions on how to perform the intended behavior [32,37,39,45,49,51-53,57,60,63] or suggestions for behavior change [45,48,63]. Some suggestion messages were of a motivational nature [32,53], such as “you have taken more rest, please go for a walk” [53]. Apart from one study [53], the *goal-setting* component was usually personalized [32,39,49,51,52,60]. Finally, goals were either assigned to the user [32,37,41,51,53,60] or the user could choose personal goals [35,39,49].

##### Design of the Persuasive eCoaching Components and Effectiveness on Health Outcomes

When comparing the use of *reduction* among the 3 categories of effectiveness, 6 out of 7 [32,39,41,51,52,60] high effective interventions used reduction by setting short-term goals to eventually reach the ultimate long-term goal. This was not done in the other, low effective or ineffective, interventions.

When comparing the effectiveness and the application of *personalization*, it became apparent that 4 out of 9 high effective interventions [32,39,51,60] used personalization to *set goals*, whereas none of the low effective and only one ineffective intervention [52] applied this strategy. Moreover, high effective studies were the only ones to personalize goals by means of self-tracking data [32,51,60]. In addition, differences were observed in the number of personalized components in the interventions, with 5 out of 9 high effective studies [32,39,41,51,60] personalizing 2 or more components in comparison with 1 out of 3 low effective [57] and 1 out of 3 ineffective interventions [52].

It was observed that 2 out of 3 high effective interventions that applied *reminders* used those reminders to ask the participant to input behavioral data into the technology [51,60], whereas ineffective and low effective interventions only used reminders on changing health behavior [35,37,44,52,53,57].

No clear pattern was observed between the 3 categories of effectiveness on health outcomes and the specific design of the simulation and suggestion components.

Other persuasive eCoaching key components for which patterns were observed regarding their effectiveness on health outcomes were the inclusion of *praise* messages [32,39,51,60] and *tunneling* by providing advice based on how well the participant changed the desired behavior [41,45,63]. These components were only ever applied in high effective interventions.

##### Self-tracking Key Components

The *validity* component was applied in 21% (3/14) of the interventions [32,39,45] that showed effectiveness on health outcomes. Based on the 50% rule, the validity of the self-tracking device is thus not considered to be a key component.

**Table 3:** Interventions’ persuasive eCoaching elements, ordered by their effect in terms of health outcomes.

Persuasive eCoaching category		Effective interventions (n=14) n (%)	Total number of studies evaluating effect on health outcomes (n=17) n (%)
**Primary task support**			
	Reduction	7 (50)	8 (47)
	Tunneling	4 (29)	5 (29)
	Tailoring	3 (21)	4 (24)
	Personalization	12 (86)	15 (88)
	Simulation	8 (57)	10 (59)
	Rehearsal	2 (14)	2 (12)
**Dialogue support**			
	Praise	4 (29)	4 (24)
	Rewards	2 (14)	3 (18)
	Reminders	7 (50)	9 (53)
	Suggestion	11 (79)	13 (76)
	Similarity	2 (14)	2 (12)
	Liking	2 (14)	2 (12)
	Social role	0 (0)	1 (6)
**System credibility support**			
	Trustworthiness	1 (7)	1 (6)
	Expertise	1 (7)	1 (6)
	Surface credibility	0 (0)	1 (6)
	Real-world feel	1 (7)	1 (6)
	Authority	0 (0)	0 (0)
	Third-party endorsement	0 (0)	0 (0)
	Verifiability	0 (0)	0 (0)
**Social support**			
	Social support in general	2 (14)	3 (18)
	Social learning	0 (0)	0 (0)
	Social comparison	0 (0)	0 (0)
	Normative influence	1 (7)	1 (6)
	Social facilitation	0 (0)	1 (6)
	Cooperation	0 (0)	0 (0)
	Competition	0 (0)	0 (0)
	Recognition	0 (0)	0 (0)
**Other**			
	Educational coaching	6 (43)	7 (41)
	Goal-setting	10 (71)	13 (76)

##### Design of the Self-Tracking Components and Effectiveness on Health Outcomes

When comparing the self-tracking *device* applied by effect on health outcomes, it was observed that accelerometers were only applied in the high effective interventions [41,45,49] and the low effective interventions [37,53,57], whereas not at all in the ineffective interventions. In addition, the most intensive *effort* was asked from participants in high effective interventions to input data into the technology. To illustrate, the low effective and ineffective interventions mostly applied uploading data [35,57] or automatic transfer of the data to the technology [43,52,53]. In the high effective interventions, participants were asked for a more intensive approach than uploading data or doing nothing, such as sending a daily message with steps to the technology [32,48,51,60]. The latter was also applied in one low effective intervention [48]. Although the validity component was not identified as a key component based on the 50% rule, only in high effective studies good *validity* and reliability of the device were described [32,39,45].

##### Other Intervention Key Components

With respectively 71% (10/14) and 50% of the effective interventions applying *integration of self-tracking and persuasive eCoaching* [32,37,39,41,45,48,49,51,53,61] and *results from other research applied* [32,39,49,51,60,61,63], these components were identified as key components. The percentages of the other components were 29% (4/14) for *theory applied* [32,39,49,63], 14% (2/14) for *design testing* [39,51], and 0% for *cocreation*.

##### Design of the Other Intervention Key Components

The design of *self-tracking and persuasive eCoaching integration* usually involved the use of self-tracking data to provide feedback [32,37,41,45,48,49,51,53,60,63]. Some studies also used self-tracking data to set goals [32,51,60]. The following *results from other research* were used in intervention design: the application of a known protocol [51,60,63], methods that were evaluated as effective [32,49,61], and components from healthy lifestyle interventions that were evaluated as effective [39,43].

##### Design of the Other Intervention Components and Effectiveness on Health Outcomes

Ineffective interventions applied less intensive *implementation* strategies such as brief tutorial [35], instructions on paper [43], or nothing [52] in comparison with the high and low effective interventions, which used mostly face-to-face instructions [32,37,39,49,51,57,61,63].

#### Key Components for Usability

##### Persuasive eCoaching Key Components

An overview of all key components for usability can be found in Multimedia Appendix 4. The most apparent key components for usability are described below. In line with the key components for a positive effect on health outcomes, a pattern was observed between the following key components and a positive effect on usability:

- *Reduction* to simplify the performance of behavior [36,50,62]. In addition, participants found it useful to be able to set short-term goals [46,58]. They believed that it could contribute to their motivation [46,58]. In addition to the similarities with key components for health outcomes, users also appreciated the provision of means to simplify their performance of the behavior [58,62].
- *Personalization* of goals [50,56,58,59]. For the most part, users appreciated the ability to set personal goals because it fosters the observation of progress [50,58,59].
- *Praise* messages [42,47,55,59,62]. However, praise might require a different design for men and women, since gender differences were observed, with women appreciating praise more than men [59].
- *Reminders* were perceived useful by most [35,42,47,50]. However, the timing and frequency of the reminders are of importance to avoid annoyance, feelings of being checked up on, or guilt for not reaching the goal [42,47,50,57,59,62]. One study’s participants expressed a preference for reminders to upload or enter data into the technology [47].
- *Simulation* to observe progress [33-35,47,50,55,59,62]. Users particularly appreciated visualization of self-tracking data to observe progress toward their goals [33-35,47,50,55,59,62]. A clear overview with only a few important features displayed was preferred overall [55,62]. However, people following physical activity guidelines preferred more detailed information [62].

In contrast to studies evaluating the effect on health outcomes, where results on *personalization* were mostly observed for the personalization of content, the participants’ concerns regarding usability were mostly about the ability to set technical features such as the timing of the message, password protection, and layout. For example, not all participants were concerned about the safety of their self-tracking data [46,50,56,59], and some found that password protection interferes with the technology’s usability [50,59]. In addition, participants would like to be able to decide whom to share data with [46,50]. These aspects also relate to the *trustworthiness* component. As for personalization of content, users acknowledged personalization as a practical solution [50] to account for the differences that existed among the various groups of users and even within groups of users [34,36,50,62]. Participants themselves also expressed a desire for the personalization of content [34,47,57,58]. Some participants felt that it would be meaningful to take personalization to the next level by using data mining to enable context-sensing and observe trends and patterns in personal data [46,47,50,59], which is also a form of *reduction*. However, others felt such extensive personalization would be unreliable, artificial, or unnecessary [47,50,59].

The *social support* component was rated negatively by most participants [34,50,59,62]. However, it appears that acceptability of social support was higher when receiving support via the technology from close friends, family, or peers [50,59,62]. However, a few participants did not like the idea of receiving support from family members, as they had previous negative experiences with support from family during behavior change [58]. In contrast, acceptability of social support was lower when the intervention used social media platforms open to everyone, such as Facebook [59,62].

In terms of users’ perspective on *educational coaching*, the fact that most users had already been trying to change their behavior for quite some time and were already familiar with much of the information on the subject should be taken into account [47,58].

##### Self-Tracking Key Components

Overall, it was apparent from the studies on usability that users had a positive attitude regarding the self-tracking of behavioral outcomes [34,46,47,51,59,63]. One positive aspect mentioned by participants was that performing self-tracking increased their awareness [36,46,47,49,62].

In line with key components for effect on health outcomes, the *validity* of the device was perceived as important among users [46,56,59]. In addition, one publication reported on users’ willingness to put in more *effort* if they felt doing so was justified by its added value [50]. Overall, most participants had a favorable attitude toward the automatic tracking of behavioral outcomes [46,59,62], although basic data entry was also perceived as acceptable [46,50,62].

As for the *measurement* component, self-tracking was found to have a potential demotivating effect when users were unable to capture all personally relevant data using self-tracking devices (eg, the use of an accelerometer when walking or running was not in fact their most common physical activity) [33,55,62].

##### Other Intervention Key Components

In line with key components for effect on health outcomes, participants acknowledged the advantages of *the integration of self-tracking and persuasive eCoaching* [32,34,35,46, 51,56,59,62] and believed that an intervention incorporating this combination could successfully motivate or change behavior [36,40,42,47,54]. In addition, participants considered it useful to receive instructions on how to use the intervention [42,56].

Furthermore, participants preferred the use of mobile phones for intervention delivery to delivery via the computer [46,50,55,58,59,62]. One advantage of mobile phone apps the participants named was the ability to use the intervention whenever they wanted [36,47].

Most participants reported that it was preferable to have access to a health care professional on top of using the automated intervention [46,47,54,55,58,59]. Even though health care professionals were negative about the provision of feedback [55], they did see the advantage of such interventions to supplement in-person sessions, as it might increase their ability to anticipate and better understand the process of behavior change among clients [40,55].

#### Key Components for Adherence

No key components for adherence could be identified based on information about intended usage, as these results were only sparingly presented [40,41,45,47,51,53,60,63]. Out of the 8 studies that did present results on intended usage, only 6 presented data on the intended usage of the self-tracking component and not the intervention as a whole [41,45,47, 51,60,63].

Based on participants’ opinions, the following key components were identified: the *personalization* component, as users believed that personally relevant advice could increase adherence [46,59,62]; and the *design testing* component, as users said that adherence declined when a problem occurred while using the intervention [36,62].

## Discussion

### Findings

This scoping review aimed to identify key components of self-tracking and persuasive eCoaching in automated healthy lifestyle interventions that contribute to the effectiveness on health outcomes, usability, and adherence.

#### Key Components for Effect on Health Outcomes

A pattern was observed between the following key components and a positive effect on health outcomes: *reduction*, *personalization*, *simulation*, *suggestion*, *goal-setting*, *praise*, use of *valid* wearables and specifically accelerometers, *integration of self-tracking and persuasive eCoaching*, use of *results from other research* to inform design, and provision of face-to-face instructions during *implementation* of the intervention. A pattern was also observed between more *effort* by the participant to input the self-tracking data in the intervention and more effect on health outcomes. For the following key components, it appears that a specific design is required for the component to have a positive influence on health outcomes: *reduction* by setting short term goals to eventually reach long-term goals, *personalization* of goals using self-tracking data, personalization of multiple components, *tunneling* by provision of feedback based on how well the user changed their behavior, and *reminders* to input data into the technology.

Similar to this scoping review’s results, other recent reviews on eHealth also observed the contribution to effectiveness of reminders [68,69], personalization [70], and integration of self-tracking and persuasive eCoaching [68,71]. In addition, one review found that less persuasive technologies were extracted from the system credibility support and social support categories [14]. This could either indicate that designers do not pay enough attention to these categories of persuasive technologies or that these components are often omitted in the description of the technology in publications. If the first is true, this might have consequences for the effectiveness of the intervention. For the system credibility support category, Harris et al [72] found that users engaged less with the technology when credibility was lacking. Neglect of the social support category will be addressed in further detail below.

Support for the importance of reminders can be found in the reviews by Neff and Fry [69] and Bardus et al [68]. However, more knowledge is needed about the reminder component’s specific design and effectiveness [69,73,74]. This scoping review diminishes this research gap to some extent by indicating that sending reminders to signal self-tracking could increase effectiveness, which is consistent with the findings of one RCT study [75]. Reminders regarding behavior change appeared to be less effective. One possible explanation for this is that reminders regarding behavior change remind users of their failure to change behavior [76]. Knowledge about other aspects of the reminder component is also of importance, such as the proper frequency, timing, and the way in which users should be notified by reminders (eg, visual or audible cues). One review mentioned that a frequency of one reminder per day should be considered [71]. Another study found that sending event-based reminders (such as after breakfast) were more effective for health behavior change than time-based reminders (such as at a specified time) [74].

Requiring more effort from the participant to input data into the intervention appears to have a positive influence on interventions’ effect on health outcomes. One explanation for this, from the review by Kelders et al [15], is that if more action is required from the participant, it might make the participant more engaged with the intervention. In addition, one study on usability [50] mentioned that participants are willing to devote a higher level of effort (eg, manually entering data), as long as the effort is balanced by its added value (eg, more personally relevant feedback).

#### Key Components for Usability

Several key components were identified for a positive effect on usability. The most apparent key components for a positive effect on usability are described below. Similar to key components for effect on health outcomes, key components for usability were inclusion of *reduction*, *personalization*, *reminders*, *praise*, *simulation* to observe progress, use of *valid* wearables, *integration of self-tracking and persuasive eCoaching*, provision of face-to-face instructions during *implementation* of the intervention, and requesting more *effort* from the participant for input of self-tracking data into the technology. As for key components for effect on health outcomes, participants deemed the following specific key component designs to be preferable: *reduction* by setting short term goals to eventually reach long-term goals, *personalization* of goals, and *reminders* to input data into the technology. Participants also considered the frequency and timing of reminders to be important to avoid annoyance and acknowledged the advantages of personalizing several aspects of the design. Furthermore, a negative attitude toward *social support* was observed. To increase the acceptability of social support, designs should include the provision of social support via peers, close friends or family, and eliminate the use of social media platforms open to everyone. It was apparent that participants appreciated the delivery of the intervention via a mobile phone. On top of that, participants and health care professionals liked the idea of using the automated intervention as a supplement to in-person sessions.

A recent qualitative review on engagement with digital health interventions obtained mostly similar results [76]. Similarities include the importance of reminders, personalization, the ability to use the intervention 24/7, a suitable supplement to in-person sessions, and provision of reduction to observe trends and patterns. A preference for automated self-tracking was also observed in line with our results. However, our scoping review also uncovered that users may also be willing to accept having to put in some level of effort for self-tracking. It is also worth noting that other studies on usability of eHealth have indicated that a positive attitude exists concerning self-tracking [77-79], inclusion of praise [80], personalization of goals [80,81], the ability to observe progress [80], use of validity-tested devices [78], and that not everyone is concerned with privacy issues [82].

The studies presenting results on usability are a way for us to learn what is most valued or noticed by users about healthy lifestyle interventions combining self-tracking and persuasive eCoaching. A few observations can be made about this topic. First, we observed that the studies evaluating an intervention for effect on health outcomes were mostly focused on the personalization of intervention content, whereas studies on usability were mostly focused on the personalization of technical features. This could indicate that the importance of technical feature personalization for users is not given sufficient attention during intervention development. On the other hand, it is also possible that results on technical feature personalization were simply described less in studies evaluating the effect on health outcomes in comparison with studies on usability. One element of usability is the ease of use of a technology and studies on usability might, therefore, focus more on technical features.

Second, social influence and support are often components of traditional health behavior change models recognized in earlier research as effective for changing behavior [83-85]. But even though social support might be effective in changing behavior, we also observed an unfavorable attitude in many participants toward social support. In addition, to our knowledge, no study found strong evidence for the contribution that social support might make in automated interventions towards improving health behavior [86]. This could be explained by the low usage of the social support component observed in publications included in this review [38,58], as well as in other research [20,87]. As advocated by Riley et al [88], applying traditional health behavior change models might not be the best fit for healthy lifestyle interventions via technology due to their interactive and adaptive character. The social support component probably requires different strategies via technology than via face-to-face provision of social support.

Third, it was observed that not everyone appreciated a high level of *personalization* of feedback messages via data mining in order to discover patterns. The observation of patterns helps users become aware of their way of living and the consequences thereof. Although awareness is a first important step in behavior change [14], some people might prefer not to discover patterns they were not aware of.

Finally, this review’s results show that participants would appreciate the ability to consult a health care professional during the intervention. However, health care professionals seemed less open to this. Including consultation by a health care professional would also be a costly way of increasing usability. In terms of their effect on health outcomes, earlier research found automated interventions to be as effective as interventions that include human coaching [15]. Furthermore, this scoping review observed positive effects on health behavior change not only in blended coaching interventions but also in fully automated interventions. Therefore, including human coaching is probably not an essential component. This viewpoint is also supported by previous research [89].

#### Key Components for Adherence

Few studies included in this review described adherence that concerned the intended usage of the intervention. When intended usage was described, most of the information dealt with the self-tracking part and not the intervention as a whole. In addition, most studies that presented data on the usage of specific components did not state the intended usage in advance. This was also observed in another review [15]. Key components for a positive effect on adherence could therefore only be identified based on participants’ expectations. According to users, adherence could be increased by the *personalization* of content and the performance of *design testing* to eliminate problems during usage of the intervention. Personalization of content has been recognized in another review as a facilitator for adherence [70].

### Recommendations for Future Design and Research

First, the key components identified in this scoping review are identified as separate components. However, components might interact with each other and could lead to different outcomes than those anticipated based on knowledge of single components. One review on e-mental health interventions indicated that some combinations of persuasive technology components do indeed differ in terms of their synergy [90]. Further research is recommended to identify the most effective combination and dosage of the key components in healthy lifestyle interventions combining self-tracking and persuasive eCoaching. To date, most studies evaluating eHealth designs apply the traditional RCT method [91]. However, RCTs are often too time-consuming to keep up with the speed of technological developments and can explain little about separate elements and their contribution to effectiveness [91-93]. Riley and Rivera [94], Hekler et al [93], and Pham et al [91] advocated for new strategies to identify and design effective intervention components—“opening the black box.” Technology can provide a meaningful contribution to such discoveries, for example, by means of the “Model Predictive Control” [94]. This strategy changes the intensity and combination of intervention components on a daily basis by using the monitoring data provided by participants’ responses and other contextual factors.

Another strategy is suggested by Sieverink et al [94]. This strategy does not only attempt to open the black box, it also contributes to more insight into adherence. Sieverink et al provided preliminary results for the development of a log data protocol for eHealth technologies to identify their adherence level and effect on health outcomes [94]. They suggest collecting log data on the usage and intensity of usage for specific intervention components to be able to draw conclusions regarding adherence and linking such log data with effects on health outcomes to be able to draw conclusions regarding adherence to specific components and their effects on health outcomes.

Future research and design should focus specifically on the reminder design, social support, and the observation of patterns through data mining, as different designs seemed to influence effect on health outcomes and/or usability. It would be interesting for future research to test variations on components’ designs and their effects on health outcomes and usability.

Another recommendation is the application of personalization to account for the variation in preferences between groups of participants and even within groups of participants. Besides the fact that it is a practical way to account for the existing differences between users, participants also considered personalization to be useful. In addition to the application of personalization in the design, the observed differences both among and within groups also suggest that a needs assessment is required before and during the design phase of an intervention using self-tracking and persuasive eCoaching. Although cocreation is often mentioned as an important aspect in design models [95,96], only a few publications cited in this review described anything regarding cocreation between intervention developers and the target group. The importance of cocreation has been indicated as important to increase satisfaction with the design by other reviews [67,69]. Similar to the component of cocreation, few publications described anything about the theory underpinning the design of the intervention. The main message of other reviews in the field of eHealth is that the design of current eHealth interventions is often not based on existing theory [67,69]. The use of theory has been recognized as resulting in higher effectiveness [97].

### Strengths and Limitations

One of the strengths of this research is that by applying qualitative research methods, an attempt has been made to not only describe which components might contribute to effectiveness but also which specific component design is most effective in terms of health outcomes, usability, and adherence. This is important because applying components from one theory in different interventions can result in various designs, whereas applying components from different theories can result in interventions with quite similar designs [93]. Another strength is the fact that we used both data from RCT studies and studies in real-life settings, providing a more realistic overview of the opportunities and challenges for interventions that combine self-tracking and persuasive eCoaching in practice than we would have been able to by relying only on results from RCTs.

One limitation of this scoping review is that potential biases might have influenced the results. First, publication bias could be present, indicated by the absence of negative effects reported and the higher number of high effective interventions included in this scoping review versus low effective or ineffective interventions. With results from only 3 ineffective studies, we could not come to conclusions about any one key component being more often applied in effective interventions compared to ineffective interventions. Due to this limitation, we introduced the 50% rule to identify key components. It should be mentioned that the most commonly applied components in interventions were, therefore, more likely to be identified as key components.

Second, we observed that interventions described in publications from 2013 already differ to some extent from interventions described in publications from 2016. The importance of certain identified key components for effectiveness might increase or decrease due to new technological developments. The main differences were the more frequent use of accelerometers and mobile sensors for self-tracking in newer publications and the delivery of the intervention via mobile phone in newer publications in comparison with computer in older publications. This trend is likely to continue [73,98]. An example of a key component that may become more important is *the ability to enable or disable observation of trends and patterns*. The use of mobile phone sensors enables collection of a wide spectrum of personal data. As indicated by this scoping review’s results, not every user is open to intensive data mining. As another example, the importance of applying the proper *frequency and timing* of reminders may increase *.* Mobile phone interventions can use a broader set of tools to send reminders than computer-based interventions. In addition, reminders cannot be ignored as easily due to visual or audible alerts [73].

Third, we did not make a distinction between health outcomes. It could be the case that interventions targeting a more intermediate health outcome (eg, an effect on physical activity instead of an effect on blood pressure) were more easily identified as high effective studies. Fourth, the extraction of data concerning persuasive eCoaching components is somewhat subjective, which was observed by the two researchers during data extraction comparison. Finally, we did not code intervention components from the actual interventions because technology is a rapidly evolving field of research and this would have taken a significant amount of time. Choices such as this one are characteristic of the scoping review methodology [26]. These limitations limited us in making a definite list of key components. However, we attempted to provide a first impression of key components in this relatively new field of research.

### Conclusions

To our knowledge, this scoping review provides a first overview of key components and effects on health outcomes, usability, and adherence. The following key components and their specific design both seem to influence health outcomes and usability in a positive way: *reduction* by setting short term goals to eventually reach long-term goals, *personalization* of goals, *praise*, *reminders* to input self-tracking data into the technology, use of *validity-tested* devices, *integration of self-tracking and persuasive eCoaching*, and provision of face-to-face instruction during *implementation*. In addition, health outcomes or usability were not affected when more *effort* was requested from participants to input data into the technology. Unfortunately, we were limited in our ability to identify key components for adherence. Still, one key component identified for both usability and adherence is the provision of *personalized* content. Identification of key components for adherence is highly important because adherence is a prerequisite for interventions to be effective. This scoping review provides a first overview, and future research is needed to confirm the key components identified for effect on health outcomes and usability, identify key components for adherence, and study whether the key components represent an effective combination of components.

## Appendix

Multimedia Appendix 1 Full search query per database.

Multimedia Appendix 2 Principles and examples of persuasive eCoaching components.

Multimedia Appendix 3 Overview and characteristics of included publications.

Multimedia Appendix 4 Overview of identified key components and their specific design for effects on health outcomes, usability, and/or adherence.

## Abbreviations

CeHRes roadmap: Center for eHealth Research roadmap
PSD model: persuasive system design model
RCT: randomized controlled trial
SMS: Short Message Service
