# Supplementary material for: Key Components in eHealth Interventions Combining Self-Tracking and Persuasive eCoaching to Promote a Healthier Lifestyle: A Scoping Review
Source: J Med Internet Res. 2017 Aug 1;19(8):e277. doi: 10.2196/jmir.7288 (PMC5558041; doi:10.2196/jmir.7288)
Supplement: Multimedia Appendix 1 [file jmir_v19i8e277_app1.pdf]

Multimedia Appendix 1 – Search query

Pubmed:

("quantified self" OR self-track* OR wearable* OR "activity tracker" OR "wireless body area network" OR "body sensor network" OR "motion sensor" OR accelerometer OR pedometer OR "step counter" OR "smart watch" OR "remote sensing technology" OR biofeedback OR "objective measure*" OR "self-monitor*" OR "health monitor*" OR "wireless technology" OR Telemonitoring OR "personal health records" OR "self-sensing" OR "lifelogging" OR "self-surveillance" OR "Personal informatics" OR "self-monitoring" OR "remote monitoring" OR "continuous monitoring" OR "ambulatory monitoring" OR "monitoring technology" OR "health monitoring" OR "continuous monitoring" OR "lifestyle monitoring" OR "physical activity monitoring" OR "sleep monitoring" OR "stress monitoring") AND ("persuasive technology" OR "telehealth" OR Telemedic* OR Telemedicine OR mHealth OR ehealth OR "computer-assisted therapy" OR "behavior change support system" OR captology OR "Persuasive computing" OR "remote consultation" OR teleconsultation OR "mobile health" OR "virtual coaching" OR "virtual consultation" OR etherap* OR "persuasive telehealth" OR "remote coaching" OR "persuasive communication") AND ("Health behavior" OR "health behaviour" OR "health promotion" OR "behavior change" OR "behaviour change" OR "behavioral change" OR "behavioural change" OR "behavior intervention" OR "behaviour intervention" OR "behavioral intervention" OR "behavioural intervention" OR "Health prevention" OR "lifestyle prevention" OR "lifestyle intervention" OR "lifestyle program" OR "lifestyle programme" OR "lifestyle change" OR "Health education" OR "behavior control" OR "behaviour control" OR "behavioral control" OR "behavioural control" OR "Health program" OR "health programme" OR "Health intervention" OR "Lifestyle modification" OR "healthy lifestyle" OR "physical activity" OR "sedentary lifestyle" OR "exercise" OR sleep OR "stress, psychological" OR "mental stress" OR "emotional stress" OR relaxation OR "relaxation therapy" OR "relaxation training")

PsycINFO:

(("quantified self" OR self-track* OR wearable* OR "activity tracker" OR "wireless body area network" OR "body sensor network" OR "motion sensor" OR accelerometer OR pedometer OR "step counter" OR "smart watch" OR "remote sensing technology" OR biofeedback OR "objective measure*" OR "self-monitor*" OR "health monitor*" OR "wireless technology" OR Telemonitoring OR "personal health records" OR "self-sensing" OR "lifelogging" OR "self-surveillance" OR "Personal informatics" OR "self-monitoring" OR "remote monitoring" OR "continuous monitoring" OR "ambulatory monitoring" OR "monitoring technology" OR "health monitoring" OR "continuous monitoring" OR "lifestyle monitoring" OR "physical activity monitoring" OR "sleep monitoring" OR "stress monitoring") AND ("persuasive technology" OR "telehealth" OR Telemedic* OR mHealth OR m-health OR ehealth OR e-health OR "computer-assisted therapy" OR "behavior change support system" OR captology OR "Persuasive computing" OR "remote consultation" OR teleconsultation OR "mobile health" OR "virtual coaching" OR "virtual consultation" OR etherap* OR e-therap* OR "persuasive telehealth" OR "remote coaching" OR ecoaching OR e-coaching OR "persuasive communication") AND ("Health behavior" OR "health behaviour" OR "health promotion" OR "behavior* change" OR "behaviour* change" OR "behavior* intervention" OR "behaviour* intervention" OR

“Health prevention” OR “lifestyle prevention” OR “lifestyle intervention” OR “lifestyle program” OR “lifestyle programme” OR “lifestyle change” OR “Health education” OR “behavior* control” OR “behaviour* control” OR “Health program” OR “health programme” OR “Health intervention” OR “Lifestyle modification” OR “healthy lifestyle” OR “physical activity” OR “sedentary lifestyle” OR “exercise” OR sleep OR “stress, psychological” OR “mental stress” OR “emotional stress” OR relaxation OR “relaxation therapy” OR “relaxation training”))

Scopus:

TITLE-ABS-KEY((“quantified self” OR self-track* OR wearable* OR “activity tracker” OR “wireless body area network” OR “body sensor network” OR “motion sensor” OR accelerometer OR pedometer OR “step counter” OR “smart watch” OR “remote sensing technology” OR biofeedback OR “objective measure*” OR “self-monitor*” OR “health monitor*” OR “wireless technology” OR Telemonitoring OR “personal health records” OR “self-sensing” OR “lifelogging” OR “self-surveillance” OR “Personal informatics” OR “self-monitoring” OR “remote monitoring” OR “continuous monitoring” OR “ambulatory monitoring” OR ”monitoring technology” OR “health monitoring” OR “continuous monitoring” OR “lifestyle monitoring” OR “physical activity monitoring” OR “sleep monitoring” OR “stress monitoring”) AND (“persuasive technology” OR “telehealth” OR Telemedic* OR mHealth OR m-health OR ehealth OR e-health OR “computer-assisted therapy” OR “behavior change support system” OR captology OR “Persuasive computing” OR “remote consultation” OR teleconsultation OR “mobile health” OR “virtual coaching” OR “virtual consultation” OR etherap* OR e-therap* OR “persuasive telehealth” OR “remote coaching” OR “persuasive communication”) AND (“Health behavior” OR “health promotion” OR “behavior* change” OR “behaviour* change” OR “behavior* intervention” OR “behaviour* intervention” OR “Health prevention” OR “lifestyle prevention” OR “lifestyle intervention” OR “lifestyle program” OR “lifestyle programme” OR “lifestyle change” OR “Health education” OR “behavior* control” OR “behaviour* control” OR “Health program” OR “health programme” OR “Health intervention” OR “Lifestyle modification” OR “healthy lifestyle” OR “physical activity” OR “sedentary lifestyle” OR “exercise” OR sleep OR “stress, psychological” OR “mental stress” OR “emotional stress” OR relaxation OR “relaxation therapy” OR “relaxation training”))

EMBASE:

(((quantified) NEAR/3 (self)) OR ((Activity) NEXT (tracker)) OR Wearable* OR ((Self) NEXT (track*)) OR ((Wireless) NEXT (Body) NEXT (Area) NEXT (Network)) OR ((body) NEXT (sensor) NEXT (network)) OR ((Motion) NEXT (sensor)) OR Accelerometer OR Pedometer OR ((Step) NEXT (counter)) OR ((Smart) NEXT (watch)) OR ((Remote) NEXT (sensing) NEXT (technology)) OR biofeedback OR ((objective) NEXT (measure)) OR ((self) NEXT (monitor*)) OR ((health) NEXT (monitor*)) OR ((wireless) NEXT (technology)) OR Telemonitoring OR ((personal) NEXT (health) NEXT (records)) OR ((self) NEXT (sensing)) OR lifelogging OR ((self) NEXT (surveillance)) OR ((personal) NEXT (informatics)) OR ((self) NEXT (monitoring)) OR ((remote) NEXT (monitoring)) OR ((ambulatory) NEXT (monitoring)) OR ((continuous) NEXT (monitoring)) OR ((health) NEXT (monitoring)) OR ((lifestyle) NEXT (monitoring)) OR ((physical) NEXT

(activity) NEXT (monitoring)) OR ((sleep) NEXT (monitoring)) OR ((stress) NEXT (monitoring)) OR ((monitoring) NEXT (technology))) AND (((Persuasive) NEXT (technology)) OR Telehealth OR Telemedic* OR mHealth OR eHealth OR m-health OR e-health OR ((computer) NEXT (assisted) NEXT (therapy)) OR ((Behavior) NEXT (change) NEXT (support) NEXT (systems)) OR Captology OR ((Persuasive) NEXT (computing)) OR ((remote) NEXT (consultation)) OR Teleconsultation OR ((mobile) NEXT (health)) OR ((virtual) NEXT (coaching)) OR ((virtual) NEXT (consultation)) OR ecoaching OR e-coaching OR etherap* OR e-therapy OR ((persuasive) NEXT (telehealth)) OR ((remote) NEXT (coaching)) OR ((persuasive) NEXT (communication))) AND (((Health) NEXT (behavior)) OR ((health) NEXT (behaviour)) OR ((Health) NEXT (promotion)) OR ((behavior*) NEXT (change)) OR ((behaviour*) NEXT (change)) OR ((behavior*) NEXT (intervention)) OR ((behaviour*) NEXT (intervention)) OR ((Health) NEXT (prevention)) OR ((lifestyle) NEXT (prevention)) OR ((lifestyle) NEXT (intervention)) OR ((lifestyle) NEXT (program)) OR ((lifestyle) NEXT (programme)) OR ((health) NEXT (program)) OR ((health) NEXT (programme)) OR ((lifestyle) NEXT (change)) OR ((health) NEXT (education)) OR ((behavior*) NEXT (control)) OR ((behaviour*) NEXT (control)) OR ((health) NEXT (intervention)) OR ((Lifestyle) NEXT (modification)) OR ((healthy) NEXT (lifestyle)) OR ((Physical) NEXT (activity)) OR (exercise) OR ((sedentary) NEXT (lifestyle)) OR (sleep) OR ((psychological) NEXT (stress)) OR ((mental) NEXT (stress)) OR ((emotional) NEXT (stress)) OR (relaxation) OR ((relaxation) NEXT (therapy)) OR ((relaxation) NEXT (training)))
