# Supplementary material for: Key Components in eHealth Interventions Combining Self-Tracking and Persuasive eCoaching to Promote a Healthier Lifestyle: A Scoping Review
Source: J Med Internet Res. 2017 Aug 1;19(8):e277. doi: 10.2196/jmir.7288 (PMC5558041; doi:10.2196/jmir.7288)
Supplement: Multimedia Appendix 2 [file jmir_v19i8e277_app2.pdf]

Multimedia Appendix 2

Table. Overview of persuasive eCoaching components.[a]

Persuasive eCoaching component	Principle citations	Example
**Primary task support**	Components that support the users in “the carrying out of the user’s primary task” [9].	
Reduction	“A system that reduces complex behavior into simple tasks helps users perform the target behavior, ant it may increase the benefit/cost ratio of a behavior” [9].	Setting incremental daily step goals to eventually reach the long term goal.
Tunneling	“Using the system to guide users through a process of experience provides opportunities to persuade along the way” [9].	Only when participants succeeded to reach the current daily step goal, they are provided with access to information on how to continue their progress.
Tailoring	“Information provided by the system will be more persuasive if it is tailored to the potential needs, interests, personality, usage context, or other factors relevant to a user group” [9].	The layout of a mobile applications is adjusted based on user’s gender.
Personalization	“A system that offers personalized content or services has a greater capability to persuade” [9].	Based on a user’s weight loss, the dietary recommendations will be adjusted.
Simulation	“Systems that provide simulations can persuade by enabling users to observe immediately the link between cause and effect” [9].	A graph is showing the successfully completed exercise tasks per day in relation to the increase in daily steps.
Rehearsal	“A system providing means with which to rehearse a behavior can enable people to change their attitudes or behavior in the real	In a weight management intervention, video instructions are provided on how to cook a low-fat meal.

	world" [9].	
**Dialogue support**	Components "related to implementing computer-human dialogue support in a manner that helps users keep moving towards their goal or target behavior" [9].	
Praise	"By offering praise, a system can make users more open to persuasion" [9].	Participants receive a praise message when they reach their weekly weight-loss goal.
Rewards	"Systems that reward target behaviors may have great persuasive powers" [9].	Participants can collect coins when they completed an exercise and return them into discounts for fruit and vegetables.
Reminders	"If a system reminds users of their target behavior or usage of the system, the users will more likely achieve their goals" [9].	The system reminders the participant to upload their weekly accelerometer data into the system.
Suggestion	"System offering fitting suggestions will have greater persuasive powers" [9].	A user receives a message suggesting to take the bicycle to work instead of the car.
Similarity	"People are more readily persuaded through systems that remind them of themselves in some meaningful way" [9].	The system sends out reminders at convenient moments in time for the user.
Liking	"A system that is (visually) attractive for its users is likely to be more persuasive" [9].	The system provides a weekly funny fact struggles with physical activity behavior change.
Social role	"If a system adopts a social role, users will more likely use it for persuasive purposes" [9].	A virtual coach greets the person before they start the coaching session of a mobile application.
**System credibility support**	Components that support the design "to be more credible and thus more persuasive." [9]	
Trustworthiness	"A system is viewed as trustworthy will have	A mobile applications, that collects personal

	increased powers of persuasion” [9].	health data by means of a wearable device, makes a promise that the data will not be shared with third parties.
Expertise	“A system that is viewed as incorporating expertise will have increased powers of persuasion” [9].	When the system provides suggestions for weight loss, it provides links to dietary protocols.
Surface credibility	“People make initial assessments of the system credibility based on a firsthand inspection” [9].	No or only limited commercial advertisements are provided within a mobile phone application.
Real-world feel	“A system that highlights people or organization behind its content or services will have more credibility” [9].	Including contact information of the organization of development in a mobile intervention.
Authority	“A system that leverages roles of authority will have enhanced powers of persuasion” [9].	Provision of information in a mobile application about development in collaboration with health care professionals.
Third-party endorsement	“Third-party endorsements, especially from well-known and respected sources, boost perceptions on system credibility” [9].	This mobile application is approved as an eHealth intervention by the RVZ (The Council for Public Health and Health Care)
Verifiability	“Credibility perceptions will be enhanced if a system makes it easy to verify the accuracy of site content via outside sources” [9].	Links are provided to the original source of the content used in the mobile application
**Social support**	Components in the design that “motivates users by leveraging social influence” [9].	
Social support in general	The system motivates the user by leveraging social influence which cannot be placed under specific components	Users are able to share information about their jogging routes without knowing the goal behind it: obtaining

	such as social learning or social facilitation as the goal of the specific social support component is unknown.	recognition, social learning or maybe just see that others are performing the behavior along with them (social facilitation).
Social learning	“A person will be more motivated to perform a target behavior if (s)he can use a system to observe others performing the behavior” [9].	A user presents the activities he/she performed which has led to obtainment of a physical activity goal.
Social comparison	“System users will have a greater motivation to perform the target behavior if they can compare their performance with the performance of others” [9].	The average number of steps taken from a user with similar characteristics can be observed and compared with the user’s own average number of steps.
Normative influence	“A System can leverage normative influence or peer pressure to increase the likelihood that a person will adopt a target behavior” [9].	Before and after pictures are presented of people who succeeded to lose weight.
Social facilitation	“System users are more likely to perform target behavior if they discern via the system that others are performing the behavior along with them” [9].	A blog can be posted on an online platform where people can find others with similar goals.
Cooperation	“A system can motivate users to adopt a target attitude or behavior by leveraging human beings’ natural drive to co-operate” [9].	When 90% of a group reached their step goal that they, the whole group receives virtual rewards.
Competition	“A system can motivate users to adopt a target attitude or behavior by leveraging human beings’ natural drive to compete” [9].	Users will be ranked within a group of users based on their steps taken to observe who has performed the most physical activity during a week.
Recognition	“By offering public recognition for an individual or group, a	Users can post their accomplishment and receive “likes” from

	system can increase the likelihood that a person/group will adopt a target behavior" [9].	other users.
**Other**		
Goal-setting	A system can motivate users to perform the target behavior by setting goals (both short and long term). It sets the focus and, by means of self-tracking data, users can objectively see how they progress.	By means of self-tracking data, a daily step goal will be set for the user with 10% increase of the average steps per day from the past week.
Educational coaching	A system can provide knowledge about the target behavior and causes and effects which could persuade the user in performing the target behavior.	A video explains why it is important for office workers to perform a few minutes of moderate physical activity per hour.
Feedback	The system provides feedback which cannot be placed under specific components such as personalization, praise, or suggestions.	A feedback message is send after participants upload self-tracking data into the system notifying that the data is received.

[a] Principles are cited from the article by Oinas-Kukkonen and Harjumaa [9] with exception of the components social support in general, goal-setting, educational coaching and feedback. These four components are added for the purpose of this review study.
