# Supplementary material for: Key Components in eHealth Interventions Combining Self-Tracking and Persuasive eCoaching to Promote a Healthier Lifestyle: A Scoping Review
Source: J Med Internet Res. 2017 Aug 1;19(8):e277. doi: 10.2196/jmir.7288 (PMC5558041; doi:10.2196/jmir.7288)
Supplement: Multimedia Appendix 3 [file jmir_v19i8e277_app3.pdf]

Multimedia Appendix 3

Table. Summary of included publications.

Study number	Evaluating effectiveness on:	Study characteristics
**Study 1**	Health outcomes	**Title:** mHealth Physical Activity intervention: A Randomized Pilot Study in Physically Inactive Pregnant Women<br>**Authors (year):** Choi et al. (2015) [36]<br>**Study design:** pilot RCT<br>**Objective:** To test a 12-week mobile health (mHealth) physical activity intervention for feasibility and potential efficacy.<br>**Participants:** women between 10 and 20 weeks of gestation and with a sedentary lifestyle (n=30)<br>**Country:** United States<br>**Effectiveness:** low effective<br>**Blended coaching:** no
**Study 2**	Health outcomes and adherence<br>**Title:** A Novel Diabetes Prevention Intervention Using a Mobile App A Randomized Controlled Trial With Overweight Adults at Risk<br>**Authors (year):** Fukuoka et al. (2015) [59]<br>**Study design:** RCT<br>**Objective:** To examine the feasibility and efficacy of a diabetes prevention	

	intervention combined with a mobile app and pedometer in overweight adults at risk for type 2 diabetes.<br>**Participants:** overweight adults with diatebes type 2 (n=60)<br>**Country:** United States<br>**Effectiveness:** high effective<br>**Blended coaching:** yes	
**Study 3**	Health outcomes	**Title:** Effectiveness of a smartphone application to promote physical activity in primary care: the SMART MOVE randomised controlled trial<br>**Authors (year):** Glynn et al. (2014) [60]<br>**Study design:** RCT<br>**Objective:** To evaluate the effectiveness of a smartphone application (app) to increase physical activity in primary care.<br>**Participants:** active Andriod smartphone user aged 16 years and older (n=90)<br>**Country:** West of Ireland<br>**Effectiveness:** high effective<br>**Blended coaching:** no
**Study 4**	Health outcomes	**Title:** A Life-Style Physical Activity Intervention and the Antibody Response to Pneumococcal Vaccination in Women<br>**Authors (year):** Long et al. (2013) [43]<br>**Study design:** RCT<br>**Objective:** To assess whether a life-style physical activity intervention improved antibody response to a pneumococcal vaccination in sedentary middle-aged women.<br>**Participants:** sedentary woman between 35 to 65 years old (n=89)<br>**Country:** United Kingdom<br>**Effectiveness:** low effective<br>**Blended coaching:** yes

**Study 5**	Health outcomes and adherence	**Title:** Effects of a Web-Based Personalized Intervention on Physical Activity in European Adults: A Randomized Controlled Trial<br>**Authors (year):** Marsaux et al. (2015) [44]<br>**Study design:** 4-arm RCT<br>**Objective:** To evaluate the effect of different levels of individually tailored advice on physical activity.<br>**Participants:** adults not following a prescribed diet or adults without altered nutrition requirements because of a medical condition (n=1607)<br>**Country:** The Netherlands<br>**Effectiveness:** high effective<br>**Blended coaching:** yes
**Study 6**	Health outcomes	**Title:** Cell Phone Intervention for You (CITY): A Randomized, Controlled Trial of Behavioral Weight Loss Intervention for Young Adults Using Mobile Technology<br>**Authors (year):** Svetkey et al. (2015) [51]<br>**Study design:** RCT<br>**Objective:** To determine the effect on weight of two mobile technology-based (mHealth) behavioral weight loss interventions in young adults.<br>**Participants:** overweight/obese participants aged between 18- to 35-year-olds (n=365)<br>**Country:** United States<br>**Effectiveness:** ineffective<br>**Blended coaching:** no
**Study 7**	Health outcomes and adherence	**Title:** A telerehabilitation intervention for patients with Chronic Obstructive Pulmonary Disease: a randomized controlled pilot trial<br>**Authors (year):** Tabak et al. (2013) [52]<br>**Study design:** pilot RCT<br>**Objective study:** First, to investigate the effects of a telerehabilitation intervention on health status and activity level of patients with COPD, compared to usual care. Second, to investigate how patients comply with the intervention and whether compliance is related to treatment outcomes.<br>**Participants:** patients with a clinical diagnosis of Chronic Obstructive Pulmonary Disease (n=34)<br>**Country:** The Netherlands<br>**Effectiveness:** low effective<br>**Blended coaching:** no
**Study 8**	Health outcomes and usability	**Title:** An Adaptive Physical Activity Intervention for Overweight Adults: A Randomized Controlled Trial

		**Authors (year):** Adams et al. (2013) [31]<br>**Study design:** RCT<br>**Objective study:** To test an adaptive intervention for PA based on Operant and Behavior Economic principles and a percentile based algorithm.<br>**Participants:** overweight and inactive adults between 18 and 65 years old (n=20)<br>**Country:** United States<br>**Effectiveness:** high effective<br>**Blended coaching:** no
**Study 9**	Health outcomes and usability	**Title:** Automated interventions for multiple health behaviors using conversational agents<br>**Authors (year):** Bickmore et al. (2013) [34]<br>**Study design:** 4-arm RCT and qualitative: semi-structured interviews<br>**Objective study:** To test an automated health counselor agent which was designed to promote both physical activity and fruit and vegetable consumption through a series of simulated conversations with users on their home computers.<br>**Participants:** adults somewhat motivated to change health behavior (precontemplation or contemplation phase) (n=122)<br>**Country:** United States<br>**Effectiveness:** ineffective<br>**Blended coaching:** no
**Study 10**	Health outcomes and usability	**Title:** The Effectiveness of Mobile Phone-Based Care for Weight Control in Metabolic Syndrome Patients: Randomized Controlled Trial<br>**Authors (year):** Oh et al. (2015) [47]<br>**Study design:** RCT<br>**Objective:** To evaluate the effect of SmartCare services on weight loss compared to the effects of existing outpatient treatments in obese patients.<br>**Participants:** obese patients with metabolic syndrome (n=422)<br>**Country:** South Korea<br>**Effectiveness:** high effective<br>**Blended coaching:** yes
**Study 11**	Health outcomes and usability	**Title:** Effectiveness of a web-based, computer-tailored, pedometer-based, physical activity intervention for adults: A cluster randomized controlled trial<br>**Authors (year):** Compernelle et al. (2015) [38]<br>**Study design:** RCT and survey<br>**Objective study:** To evaluate the effectiveness of a

		computer-tailored, pedometer-based, PA intervention in working adults.<br>**Participants:** Dutch-speaking “white-collar” employees between 18 and 65 years old (n=274)<br>**Country:** Belgium<br>**Effectiveness:** high effective<br>**Blended coaching:** no
**Study 12**	Health outcomes, usability, and adherence	**Title:** Medium-Term Effectiveness of a Comprehensive Internet-Based and Patient-Specific Telerehabilitation Program With Text Messaging Support for Cardiac Patients: Randomized Controlled Trial<br>**Authors (year):** Frederix et al. (2015) [40]<br>**Study design:** RCT and qualitative: interviews<br>**Objective:** To assess the medium-term effectiveness of an Internet-based, comprehensive, and patient-tailored telerehabilitation program with short message service (SMS) texting support for cardiac patients.<br>**Participants:** cardiac rehabilitation patients (n=140)<br>**Country:** Belgium<br>**Effectiveness:** high effective<br>**Blended coaching:** no
**Study 13**	Health outcomes and usability	**Title:** Physical Activity Loyalty Cards for Behavior Change A Quasi-Experimental Study<br>**Authors (year):** Hunter et al. (2013) [42]<br>**Study design:** two-arm quasi-experimental design and survey<br>**Objective:** To investigate the effectiveness of financial incentives to encourage adults to undertake more PA, measured using a novel objective PA tracking system.<br>**Participants:** employees in a workplace setting (n=406)<br>**Country:** Northern Ireland<br>**Effectiveness:** ineffective<br>**Blended coaching:** no
**Study 14**	Health outcomes and usability	**Title:** Automated Personalized Feedback for Physical Activity and Dietary Behavior Change With Mobile Phones: A Randomized Controlled Trial on Adults<br>**Authors (year):** Rabbi et al. (2015) [48]<br>**Study design:** RCT, survey, and qualitative: semi-structured interviews<br>**Objective:** To investigate the technical feasibility of implementing an automated feedback system, the impact of the suggestions on user physical activity and eating behavior, and user perceptions of the automatically generated suggestions.

		**Participants:** participants motivated to self-monitor and improve their fitness (n=17)<br>**Country:** United States<br>**Effectiveness:** high effective<br>**Blended coaching:** no
**Study 15**	Health outcomes, usability, and adherence	**Title:** Daily Text Messaging for Weight Control Among Racial and Ethnic Minority Women: Randomized Controlled Pilot Study<br>**Authors (year):** Steinberg et al. (2013) [50]<br>**Study design:** pilot RCT<br>**Objective:** First, to evaluate the feasibility of a text messaging intervention for weight loss among predominantly black women. Second, to evaluate the effects of the intervention on weight change relative to an education control arm.<br>**Participants:** predominantly black and obese women (n=50)<br>**Country:** United States<br>**Effectiveness:** high effective<br>**Blended coaching:** no
**Study 16**	Health outcomes, usability, and adherence	**Title:** The Efficacy of a Daily Self-Weighing Weight Loss Intervention Using Smart Scales and E-mail<br>**Authors (year):** Steinberg et al. (2013) [62]<br>**Study design:** RCT and survey<br>**Objective:** To examine the impact of a weight loss intervention that focused on daily self-weighing for self-monitoring as compared to a delayed control group among 91 overweight adults.<br>**Participants:** overweight adults (n=91)<br>**Country:** United States<br>**Effectiveness:** high effective<br>**Blended coaching:** no
**Study 17**	Health outcomes and usability	**Title:** Wearable Sensor/Device (Fitbit One) and SMS Text-Messaging Prompts to Increase Physical Activity in Overweight and Obese Adults: A Randomized Controlled Trial<br>**Authors (year):** Wang et al. (2015) [56]<br>**Study design:** RCT and survey<br>**Objective:** First, to test the effects on PA level of a technology-based intervention that delivered simple prompts using SMS text messaging in conjunction with the Fitbit One for self-monitoring. Second, to examine the usability and effects of a wearable device/sensor (the Fitbit One) on PA levels.

		**Participants:** Overweight and obese adults who were interested in increasing their PA (n=67)<br>**Country:** United States<br>**Effectiveness:** high effective<br>**Blended coaching:** no
**Study 18**	Usability	**Title:** A Persuasive and Social mHealth Application for Physical Activity: A Usability and Feasibility Study<br>**Author (year):** Al Ayubi et al. (2014) [32]<br>**Study design:** qualitative: think-aloud method and in-depth semistructured interviews<br>**Objective:** First, to identify whether the system is usable and accepted by users. Second, to reveal other issues in the deployment of this technology that contribute to an informed preparation for clinical trials.<br>**Participants:** participants aged 24-45 (n=14)<br>**Country:** United States<br>**Blended coaching:** no
**Study 19**	Usability	**Title:** Dutch young adults ratings of behavior change techniques applied in mobile phone apps to promote physical activity: A Cross-sectional Survey<br>**Authors (year):** Belmon et al. (2015) [33]<br>**Study design:** survey<br>**Objective:** First, to explore young adults' opinions regarding BCTs (including self-regulation techniques) applied in mobile phone phsycial activity apps. Second, to examine associations between personality characteristics and ratings of BCTs applied in physical activity apps.<br>**Participants:** Dutch healthy young adults (n=179)<br>**Country:** The Netherlands<br>**Blended coaching:** did not describe an intervention
**Study 20**	Usability and adherence	**Title:** Patients' experiences of using a smartphone application to increase physical activity: the SMART MOVE qualitative study in primary care<br>**Authors (year):** Casey et al. (2014) [35]<br>**Study design:** qualitative: semi-structured interviews<br>**Objective:** To explore patients' views and experiences of using smartphones to promote physical activity in primary care.<br>**Participants:** active Andriod smartphone user aged 16 years and older (n=12)<br>**Country:** West of Ireland<br>**Blended coaching:** did not describe an intervention
**Study 21**	Usability	**Title:** Development of a Weight Loss Mobile App Linked With an Accelerometer for Use in the Clinic: Usability,

		Acceptability, and Early Testing of its Impact on the Patient-Doctor Relationship<br>**Authors (year):** Choo et al. (2016) [37]<br>**Study design:** survey and observational (app usage data)<br>**Objective:** To evaluate the usability and acceptability of a newly developed mobile app linked with an accelerometer and its early effects on patient-doctor relationships.<br>**Participants:** obese individuals between 20-70 years (n=30)<br>**Country:** South Korea<br>**Blended coaching:** yes
**Study 22**	Usability and adherence	**Title:** Opportunities and Challenges for Smartphone Applications in Supporting Health Behavior Change: Qualitative Study<br>**Author (year):** Dennison et al. (2013) [58]<br>**Study design:** qualitative: focus groups<br>**Objective:** To explore young adults' perspectives on apps related to health behavior change.<br>**Participants:** university students and staff (n=19)<br>**Country:** United Kingdom<br>**Blended coaching:** did not describe an intervention
**Study 23**	Usability and adherence	**Title:** Utility and Efficacy of a Smartphone Application to Enhance the Learning and Behavior Goals of Traditional Cardiac Rehabilitation<br>**Authors (year):** Forman et al. (2014) [38]<br>**Study design:** survey and observational (app usage data)<br>**Objective:** To study the feasibility and utility of Heart Coach on an iPhone, iPad, or iPod Touch (Apple, Inc, Cupertino, CA) as an adjunct to traditional clinic-based Cardiac Rehabilitation.<br>**Participants:** staff (n=3) and patients (n=26) at South Shore Hospital's Cardiac Rehabilitation program<br>**Country:** United States<br>**Blended coaching:** yes
**Study 24**	Usability	**Title:** PREDIRCAM eHealth Platform for Individualized Telemedical Assistance for Lifestyle Modification in the Treatment of Obesity, Diabetes, and Cardiometabolic Risk Prevention: A Pilot Study (PREDIRCAM 1)<br>**Authors (year):** Gonzalez et al. (2013) [41]<br>**Study design:** survey<br>**Objective:** To assess the viability of the platform.<br>**Participants:** volunteers aged 38 ± 15 years with average skills on computer usage and no important comorbidities

		(n=15)<br>**Country:** Spain<br>**Blended coaching:** no
**Study 25**	Usability and adherence	**Title:** What features do Dutch university students prefer in a smartphone application for promotion of physical activity? A qualitative approach<br>**Author (year):** Middelweerd et al. (2015) [61]<br>**Study design:** qualitative: focus group<br>**Objective:** To explore Dutch students' preferences regarding a PA application (PA app) for smartphones.<br>**Participants:** Dutch university students aged 18-25 (n=30)<br>**Country:** The Netherlands<br>**Blended coaching:** no
**Study 26**	Usability and adherence	**Title:** Tracking Health Data Is Not Enough: A Qualitative Exploration of the Role of Healthcare Partnerships and mHealth Technology to Promote Physical Activity and to Sustain Behavior Change<br>**Authors (year):** Miyamoto (2016) [45]<br>**Study design:** qualitative: focus groups<br>**Objective:** To understand potential users' views of mHealth technology, the role this technology may have in promoting individual activity goals aimed at improving health, and the value of integrating mHealth technology with traditional health care.<br>**Participants:** working adults from an academic institution mostly with an age between 45-54 years (groups included 8-12 participants and the confirmatory group contained three participants)<br>**Country:** United States<br>**Blended coaching:** did not describe an intervention
**Study 27**	Usability and adherence	**Title:** A Text-Messaging and Pedometer Program to Promote Physical Activity in People at High Risk of Type 2 Diabetes: The Development of the PROPELS Follow-On Support Program<br>**Authors (year):** Morton et al. (2015) [46]<br>**Study design:** developing prototype and qualitative: focus groups and telephone interviews<br>**Objective:** To develope the PROPELS follow-on support program and evaluate acceptability and feasibility.<br>**Participants:** participants aged ages 39-79 years in phase 2 (n=15), participants aged 52-78 years in phase 3 (n=20), and participants from phase 2 and 3 in phase 4 (n=11)

		**Country:** United Kingdom<br>**Blended coaching:** yes
**Study 28**	Usability<br>**Title:** Identifying preferences for mobile health applications for self-monitoring and self-management: Focus group findings from HIV-positive persons and young mothers.<br>**Authors (year):** Ramanathan et al. (2013) [49]<br>**Study design:** qualitative: focus groups<br>**Objective:** To inform the design of an adaptable mobile health application we aimed to identify the dimensions and range ofuser preferences for application features by different user groups.<br>**Participants:** Two populations of mobile phone users: people living with HIV (n=29); and young mothers (n=24)<br>**Country:** United States<br>**Blended coaching:** did not describe an intervention	
**Study 29**	Usability	**Title:** The spinal stenosis pedometer and nutrition lifestyle intervention (SSPANLI): development and pilot

		**Authors (year):** Tomkins-Lane et al. (2015) [53]<br>**Study design:** pilot observational study and qualitative interviews<br>**Objective:** To develop and pilot an e-health intervention aimed at increasing physical activity and decreasing fat mass in people with lumbar spinal stenosis.<br>**Participants:** overweight or obese individuals with lumbar spinal stenosis (LSS) (n=9)<br>**Country:** Canada<br>**Blended coaching:** yes
**Study 30**	Usability	**Title:** A Mobile Phone App to Stimulate Daily Physical Activity in Patients with Chronic Obstructive Pulmonary Disease: Development, Feasibility, and Pilot Studies<br>**Authors (year):** Vorrink et al. (2016) [55]<br>**Study design:** developing prototype, survey, and qualitative: focus groups<br>**Objective:** To develop an eHealth intervention that will support patients with COPD to improve or maintain their DPA after pulmonary rehabilitation.<br>**Participants:** healthy adults (n=10) (phase 1), persons with COPD aged >40 years, living independently and completed rehabilitation (phase 2: n=3, phase 3: n=7), independent respiratory nurses (n=10) and physiotherapists (by phone) (n=2) who work with COPD patients (phase 3)<br>**Country:** The Netherlands<br>**Blended coaching:** yes
**Study 31**	Usability	**Title:** Development of an Evidence-Based mHealth Weight Management Program Using a Formative Research Process<br>**Author (year):** Waterlander et al. (2014) [57]<br>**Study design:** Online survey and qualitative: focus groups and phone interviews<br>**Objective:** To develop an evidence-based mHealth weight management program (Horizon) using formative research and a structured content development process.<br>**Participants:** participants in three focus groups (n=20), in phone interviews (n=5), and the online survey (n=120)<br>**Country:** New Zealand<br>**Blended coaching:** no
**Study 32**	Usability	**Title:** The Development of a Mobile Monitoring and Feedback Tool to Stimulate Physical Activity of People With a Chronic Disease in Primary Care: A User-Centered Design

		**Authors (year):** Van der Weegen et al. (2013) [54]<br>**Study design:** developing prototype, survey, and qualitative: semi-structured interviews<br>**Objective:** To investigate the user requirements for a tool to stimulate physical activity, embedded in primary care practice.<br>**Participants:** people with COPD or type-2 diabetes (n=15), their primary care professionals (n=16), and several experts<br>**Country:** The Netherlands<br>**Blended coaching:** yes
