# Supplementary material for: Key Components in eHealth Interventions Combining Self-Tracking and Persuasive eCoaching to Promote a Healthier Lifestyle: A Scoping Review
Source: J Med Internet Res. 2017 Aug 1;19(8):e277. doi: 10.2196/jmir.7288 (PMC5558041; doi:10.2196/jmir.7288)
Supplement: Multimedia Appendix 4 [file jmir_v19i8e277_app4.pdf]

Multimedia Appendix 4.

Table. Key components and their specific design for effects on health outcomes, usability, and/or adherence.

Key component	Design of component	Pattern observed for effect on health outcomes (H), usability (U), and/or adherence (A)	Based on results from study..
**Persuasive eCoaching**			
Reduction	1. Include reduction	H + U	H: 32, 39, 41, 49, 51, 60, 63<br>U: 36, 46, 50, 58, 62
	2. Setting short term goals	H + U	H: 32, 39, 41, 51, 60, 63<br>U: 46, 58
	3. Ability to enable or disable observation of trends and patterns	U	U: 46, 47, 50, 59
	4. Provide means to perform behavior	U	U: 58, 62
Personalization	1. Include personalization	H + U	H: 32, 37, 39, 41, 45, 48, 49, 51, 53, 57, 60, 61<br>U: 34, 36, 50, 62
	2. Personalization of goals	H + U	H: 32, 39, 51, 60<br>U: 34, 46, 47, 50, 56-59
	3. Personalization of content	U + A	U: 34, 47, 57, 58<br>A: 46, 59, 62
	4. Personalization of multiple components	H	H: 32, 39, 41, 51, 60
	5. Ability to enable or disable observation of trends and patterns	U	U: 46, 47, 50, 59
	6. Ability to adjust technical features	U	U: 42, 47,

			50, 55, 57, 59, 62
Praise	1. Include praise	H + U	H:32, 39, 51, 60<br>U: 42, 47, 55, 59, 62
	2. Account for gender differences	U	U: 59
Rewards	Include rewards (monetary or non-monetary incentives)	U	U: 36, 50, 62
Reminders	1. Include reminders	H + U	H: 32, 37, 44, 51, 53, 57, 60<br>U: 35, 42, 47, 50
	2. Reminders asking to enter/upload data into the technology	H + U	H: 51, 60<br>U: 47
	3. Timing and frequency of importance	U	U: 42, 47, 50, 57, 59, 62
Similarity	Connect with or complement existing behavior	U	U: 46, 49, 59
Simulation	Include simulation to observe progress	H + U	H: 37, 39, 48, 51, 53, 57, 61, 63<br>U: 32, 34, 35, 47, 50, 55, 59, 62
Suggestion	Include suggestion	H	H: 32, 37, 39, 45, 48, 49, 51, 53, 57, 60, 63
Social role	The technology should act as a social being to some extent	U	U: 33, 35, 62
Trustworthiness	1. Trustworthy content	U	U: 58, 59, 62
	2. Control over data sharing	U	U: 46, 50
	3. Password protection can be enabled or disabled	U	U: 50, 59
Tunneling	Provide feedback based on how well the user changed behavior	H	H: 41, 45, 63
Surface credibility	Provide a technology that is not noticed as an automated technology	U	U: 35, 57
Social support	1. Be careful with inclusion of social support (as it is rather negatively	U	U: 34, 50, 59, 62

	perceived among participants)		
	2. To increase acceptability: provide social support via peers, friends or family	U	U: 50, 59, 62
	3. To increase acceptability: Do not use social media where everybody can observe personal results	U	U: 59, 62
Cooperation	To increase acceptability of social support: incorporate cooperation among participants to work towards a similar goal	U	U: 46, 50, 59
Competition	To increase acceptability of social support: option to compete with peers or not	U	U: 59, 62
Educational coaching	Short videos which do not require large amounts of internet/mobile data	U	U: 38, 59, 62
	Account for known knowledge by participant	U	U: 47, 58
Goal setting	1. Include goal-setting	H	H: 32, 37, 39, 41, 49, 51, 53, 60, 61, 63
	2. Use self-tracking data as input for automatic goal-setting	H	H: 32, 51, 60
	3. Personalization of goals	H + U	H: 32, 39, 51, 60<br>U: 34, 46, 47, 50, 56-59
	4. Monitor progress towards goals	U	U: 33-35, 47, 50, 55, 59, 62
	5. Setting short term goals	U	U: 46, 58
Feedback	1. Provide immediate, not paternalistic, short, personalized, and positive feedback	U	U: 34-36, 39, 47, 55, 57-59, 62
	2. Timing and frequency of feedback is important	U	U: 42, 47, 50, 57, 59, 62
**Self-tracking**			
Self-tracking	Incorporate self-tracking to increase awareness	U	U: 36, 46, 47, 49, 62
Device	Accelerometer	H	H: 37, 41, 45, 49, 53, 57

Measurement	Should capture all relevant behavioral data (e.g. besides only steps for measurement of physical activity)	U	U: 33, 55, 62
The effort by the participant	1. More effort does not seem to be a barrier	H + U	H: 32, 48, 51, 60<br>U: 50
	2. Automatically tracking preferred	U	U: 46, 59, 62
Summary	1. Visualization of self-tracking data is perceived appealing	U	U: 36, 55, 56
	2. Observe progress	U	U: 33-35, 47, 50, 55, 59, 62
	3. Enable personalization of layout of the displayed summary data	U	U: 55, 62
	4. Ability to enable or disable observation of trends and patterns	U	U: 46, 47, 50, 59
Validity	Valid and reliable measurements perceived important	H + U	H: 32, 39, 45<br><br>U: 46, 56, 59
**Other intervention components**			
Applying results from other research	Use results from other research to inform the design	H	H: 32, 39, 49, 51, 60, 61, 63
Medium	1. Deliver the intervention via a smartphone	U	U: 46, 50, 55, 58, 59, 62
	2. Able to use the intervention 24/7	U	U: 36, 47
Design testing	Problems with usages of the intervention should be eliminated during the testing phase	A	A: 36, 62
Implementation	Provision of face-to-face instructions on how to use the intervention	H + U	H: 32, 37, 39, 49, 51, 57, 61, 63<br>U: 42, 56
Integration of self-tracking and persuasive eCoaching	Include integration of self-tracking and persuasive eCoaching	H + U	H: 32, 37, 39, 41, 45, 48, 49, 51, 53, 61<br>U: 32, 34-36, 39, 42, 46, 47, 51, 54, 56, 59,

			62
Blended coaching	Use automatic eHealth intervention to supplement in-person sessions	U	U: 39, 46, 47, 54, 55, 58, 59
